# SORT IT empowers oncology clinicians to boost research capacity and advance universal health coverage in India

**DOI:** 10.5588/pha.25.0039

**Published:** 2025-12-03

**Authors:** A. Seshachalam, K. Niraimathi, S.G. Raman, R. Zachariah, P. Thekkur

**Affiliations:** 1 Collaborative Medical Oncologist Group, Chennai, India;; 2 Dr GVN Cancer Institute, Trichy, India;; 3 Evidentia Research Solutions, Chennai, India;; 4 Madras Cancer Care Foundation, Apollo Cancer Institute, Chennai, India;; 5 UNICEF, UNDP, World Bank, WHO, Special Programme for Research and Training in Tropical Diseases, Geneva, Switzerland;; 6 Centre for Operational Research, International Union against Tuberculosis and Lung Disease, Paris, France.

**Keywords:** tuberculosis, cancer care, public health, hybrid training, peer mentorship

## Abstract

The Structured Operational Research and Training Initiative (SORT IT), implemented through the Collaborative Medical Oncology Group in India, strengthened oncology research capacity by training clinicians, expanding research into underserved regions, and fostering a sustainable mentorship culture. Using a descriptive evaluation (2014–2025), the programme documented growth in research outputs, mentoring, and dissemination through a Real-World Evidence Conference and Stats Decoded pre-conference workshop. Context-specific innovations – hybrid learning, mobile data capture, and faculty development – enhanced scalability. Case examples demonstrated translation of research into improved cancer screening and diagnostics. SORT IT contributed to evidence-informed cancer care and provides a replicable framework for other medical disciplines.

The Structured Operational Research and Training Initiative (SORT IT) is a partnership-based initiative led by TDR (UNICEF/UNDP/World Bank/WHO Special Programme for Research and Training in Tropical Diseases) to address the challenge of limited operational research capacity within health systems.^1^ Initially focused on infectious diseases, it has since expanded to more than 25 health domains.^2^

‘SORT IT gave us the tools and structure to turn ideas into impactful research.’

This reflection from the leadership team of the Collaborative Medical Oncology Group (CMOG) of India captures the role played by SORT IT in building research capacity among oncology clinicians. The CMOG was established in 2014 as a registered non-profit network of oncologists across South India^3^ and it began with a bold vision of generating real-world oncology data that reflect clinical practice in India. Recognising the growing burden and inequities in cancer care, CMOG adapted SORT IT to the oncology context. This intervention, which is an adaptation and implementation of the SORT IT approach within the CMOG in India, aimed to:

1. Build research capacity among oncology clinicians through structured operational research training and mentorship.
2. Foster a sustainable research culture through institutionalisation and peer mentorship, especially in underserved regions of India.

Implementation involved a year-long, milestone-driven training programme combining protocol development, data analysis, and manuscript writing, guided by experienced mentors. A logic model linked inputs (training and mentorship) to activities (protocol development, multicentre analysis) and outcomes (publications, policy/practice changes) (Table).

**TABLE.** Examples of research conducted through the Structured Operational Research and Training Initiative (SORT IT) influencing policy and practice in India.

Reference	Key findings	Policy/practice influence
4	Interim PET after two cycles predicts event-free survival better than IPS.	Facilitated the adoption of PET-based response assessment in HL across Indian centres, minimising unnecessary toxicity.
5	Halving time of BCR-ABL1 is a more accurate predictor of molecular response than single day-90 value.	This paper shifted practice from a single day-90 assessment to serial BCR-ABL1 monitoring, emphasising trend and depth of decline, and prompting CMOG centres to adopt serial testing for earlier treatment decisions.
6	Reported safe and cost-effective modifications of ATRA/ATO/DNR regimen with excellent survival in Indian high-risk APL.	Provided evidence supporting frontline ATO use in APL, leading to its incorporation into institutional and state protocols for cost-effective leukaemia care.
7	Confirmed prognostic role of interim PET2 in paediatric HL; failure to achieve CR predicted poor outcome.	Advised paediatric oncology boards on the safety and efficacy of ABVD and the integration of PET-guided risk stratification, enabling reduced radiation and fewer cycles in low-risk children.
8	Identified satisfactory and dissatisfactory factors in day-care chemotherapy services, with dissatisfaction linked to pharmacy, nursing, delays, and paediatric care.	Advocated for dedicated paediatric services, streamlined pharmacy operations, increased bed capacity, and flexible oncologist scheduling to improve efficiency and patient satisfaction. This led to workflow restructuring in centres in the state of Tamil Nadu and informed state-level discussions on scaling the Chief Minister’s Health Insurance Scheme for smoother chemotherapy delivery.

PET = positron emission tomography; ABVD = adriamycin, bleomycin, vinblastine, dacarbazine (chemotherapy regimen for Hodgkin lymphoma); IPS = international prognostic score; HL = Hodgkin lymphoma; BCR-ABL1 = breakpoint cluster region-abelson 1 fusion gene; CML = chronic myeloid leukaemia; CMOG = collaborative medical oncology group; ATRA = all-trans retinoic acid; ATO = arsenic trioxide; DNR = daunorubicin (an anthracycline chemotherapy drug); APL = acute promyelocytic leukaemia; CR = complete remission.

## GEOGRAPHIC AND TEMPORAL SCOPE OF IMPLEMENTATION

The intervention began in 2018–2019 in South India, involving 8 oncology clinicians from 12 institutions. It was later expanded in 2023–2024 to Silchar, Assam, through the Cachar Cancer Hospital and Research Centre (CCHRC), a region where 80% of patients are daily wage earners and 75% depend on subsidised care. Administrative records and publication metrics from CMOG between 2024 and 2025 were analyzed to assess training outputs, research productivity, dissemination through the Real-World Evidence (RWE) Conference, and documented examples of policy and practice influence. Sixty-two oncologists were trained through SORT IT courses and the STATS Decoded workshop – a 3-day pre-conference operational research module conducted as part of the National RWE Conference. In addition, a dedicated website for the RWE Conference^9^ was developed to facilitate knowledge sharing, networking, and wider dissemination of real-world oncology research across India.

## PURPOSE

The initiative sought to strengthen India’s oncology research ecosystem by embedding operational research within clinical practice. It aimed to transform clinician-led data generation into evidence-based decision-making, enabling real-world evidence to inform policy and improve cancer care delivery. The primary evaluative question was as follows: To what extent did the SORT IT model, implemented within CMOG, improve oncology research capacity (training outputs, research productivity, mentorship pipeline) and contribute to practice/policy changes between 2014 and 2025?

## EVALUATION AND OBSERVED OUTCOMES

A descriptive case study approach was used with predefined indicators: 1) number of clinicians trained and progressing to mentors; 2) research outputs (peer-reviewed publications, multicentre datasets); 3) dissemination metrics (RWE attendance); and 4) examples of documented practice/policy influence. Trends before (2014–2018) and after (2019–2025) the first SORT IT cohort were compared. Attribution was interpreted as ‘contributed to’ rather than causal.

### Results

Publications increased substantially, from 41 (mean 8 per year) during 2014–2018 to 155 (mean 26 per year) from 2019 onwards, indicating a marked rise in research productivity. A sustainable mentorship pipeline also emerged, as six of the eight initial trainees advanced to junior mentors and four subsequently to senior mentors by 2023. Furthermore, the RWE Conference, established in 2019, institutionalised research dissemination and peer learning across oncology centres. Attendance expanded from 352 participants in 2019 to nearly 900 in 2024, demonstrating growing national engagement and momentum in clinician-led research.

### Case examples of impact

See also the accompanying Table.

Vignette 1: integrating cancer screening into home-based palliative care.

A SORT IT–trained team at CCHRC integrated cancer education and symptom screening into palliative care visits. The approach demonstrated feasibility in a resource-limited setting with high cancer burden and low screening coverage (0.2%–0.9% population-based screening in Assam despite high tobacco use) and informed discussions on expanding early detection strategies in Northeast India.^10^

Vignette 2: frozen-section diagnosis as point-of-care testing.

In response to diagnostic delays requiring multiple hospital visits among economically disadvantaged patients, SORT IT–trained oncologists at Cachar Cancer Hospital implemented frozen-section biopsy as an alternative to routine 3-day histopathology. With a 78% cancer confirmation rate among suspected cases, same-day diagnosis reduced patient anxiety, travel costs, and lost wages. The model was subsequently incorporated into routine clinical pathways for high-probability cancer cases.^11^ No adverse effects or ethical concerns were identified. The descriptive design, while not measuring patient-level outcomes, captured consistent trends of improved research output and local practice change.

## SUSTAINABILITY

A sustainable research culture has been institutionalised within CMOG. Alumni lead research units, mentor new participants, and contribute to national datasets. The mentorship model (‘see one, do one, teach one’) created a self-sustaining faculty development pipeline, reducing reliance on external trainers. Key innovations supporting sustainability include:

### Hybrid training model

Two online modules (study protocol writing and data capture and analysis) and one in-person session (manuscript writing module) reduced costs by ∼40% while maintaining accountability and access.

### Mobile data collection

The EpiCollect5 mobile application enabled real-time, cloud-based data capture across multiple centres. Compared with paper-based systems, it was easier for busy clinicians, improved data quality, and encouraged participation.

### Leadership development

Graduates transitioned to mentors and evolved into prolific researchers, strengthening leadership renewal. Of the eight trainees from the 2018 cohort, six became junior mentors by 2020 and four progressed to senior mentors by 2023.

### Systematic dissemination

Mandatory RWE presentations fostered accountability and peer recognition, embedding research into practice.

## PUBLIC HEALTH SIGNIFICANCE

This demonstrates how structured operational research training through SORT IT can strengthen oncology systems and promote evidence-informed care, especially in low-resource and underserved regions. By transforming clinicians into researchers, it contributed to policy-relevant data generation and fostered institutional collaboration across India. This model offers a replicable framework for other medical specialties seeking to embed research culture into service delivery.
